# Membrane Technologies for Sustainable Wastewater Treatment: Advances, Challenges, and Applications in Zero Liquid Discharge (ZLD) and Minimal Liquid Discharge (MLD) Systems

**DOI:** 10.3390/membranes15020064

**Published:** 2025-02-19

**Authors:** Argyris Panagopoulos, Panagiotis Michailidis

**Affiliations:** School of Chemical Engineering, National Technical University of Athens, 9 Iroon Polytechniou St., Zografou, 15780 Athens, Greece; agmlth@hotmail.com

**Keywords:** membrane technology, desalination, brine, zero liquid discharge (ZLD), minimal liquid discharge (MLD), membranes, reverse osmosis, forward osmosis, membrane distillation, membrane advances

## Abstract

As the demand for sustainable water and wastewater management continues to rise in both desalination and industrial sectors, there is been notable progress in developing Zero Liquid Discharge (ZLD) and Minimal Liquid Discharge (MLD) systems. Membrane technologies have become a key component of these systems, providing effective solutions for removing contaminants and enabling the recovery of both water and valuable resources. This article explores recent advancements in the design and operation of ZLD and MLD systems, discussing their benefits, challenges, and how they fit into larger treatment processes. Emphasis is given to membrane-based processes, such as reverse osmosis (RO), membrane distillation (MD), and forward osmosis (FO), as well as hybrid configurations, and innovative membrane materials. These advancements are designed to address critical challenges like fouling, scaling, high energy demands, and high brine production. The article also explores exciting research directions aimed at enhancing the efficiency and durability of membrane technologies in ZLD and MLD systems, paving the way for new innovations in sustainable water management across various industries.

## 1. Introduction

The pressing issue of water shortages/scarcity has far-reaching consequences for global sustainability, with implications for humanity’s future [1,2]. A widely accepted strategy for addressing this water challenge is desalination and saline wastewater (brine) treatment, a concept that has become increasingly prevalent in various regions worldwide (e.g., in the Gulf and the Mediterranean region) [3,4]. At present, over 21,000 desalination facilities are in operation, generating a daily water output of approximately 142 million cubic meters of desalinated potable water. However, it is essential to recognize that desalination is not without its drawbacks. This process inevitably produces liquid effluent with a salinity significantly higher than that of seawater. This concentrated water stream, earmarked as ‘brine’/‘reject’/‘concentrate’, requires careful management to avoid potentially catastrophic environmental repercussions. The sheer scale of this challenge is underscored by the fact that brine effluents are generated by a multitude of industries, including dairy production, textile production, oil and gas extraction, agriculture, food manufacturing, pharmaceutical development, and leather production, among others, with a daily output of over 128 million cubic meters only from the desalination industry [5].

Brine management has undergone a shift in disposal strategies, which have been found to have deleterious environmental effects. The conventional methods of surface water discharge, evaporation ponds, deep well injection, sewer discharge, and land application are no longer deemed sustainable. Rather, the development of an approach that minimizes wastewater effluent volume and recovers valuable resources, including water, energy, salts, and chemicals, is necessary. The reclamation of water from brine can also contribute to the attainment of the sixth United Nations Sustainable Development Goal (i.e., SDG 6) [6].

In response to the pressing concerns arising from climate change and the burgeoning world population, it is imperative that we transition to a paradigm of resource stewardship that encompasses a circular economy. This endeavor necessitates a thorough re-examination and optimization of the material and resource flows within various industry sectors, with a view to fostering greater efficiency and minimizing waste. Furthermore, the development of innovative treatment processes/technologies is critical to unlock access to non-traditional, yet scarce, sources of resources, such as wastewater from industries and previously uneconomical sources, which can be harnessed in a novel and sustainable manner [7,8,9]. In an endeavor to optimize water conservation, treatment technologies and processes can be harmoniously combined into zero and minimal liquid discharge (ZLD/MLD) systems. Notably, the initial installations of ZLD systems were commissioned by power plants in Colorado, U.S., in response to the escalating salinity of the Colorado River. The primary objective of these systems is to reclaim 100% of water while eliminating wastewater by producing a solid salt. Nonetheless, in recent times, advancements in technology have led to a noteworthy shift towards the production of multiple solids rather than a single salt product. Concurrently, the MLD concept has garnered significant attention due to its comparative advantages, including reduced capital expenditures, lower energy needs, and a water reclamation target of approximately 95% [10].

Contrasting traditional thermal desalination processes, such as multi-stage flash distillation (MSF), multiple effect distillation (MED), and vapor compression (VC), which have been employed, these approaches exhibit drawbacks like substantial energy expenditure and environmental concerns, primarily encompassing the disposal of saline residues. In contrast, the main membrane-based process, reverse osmosis (RO) emerges as a more energy-efficient and eco-friendly paradigm, thereby assuming a pivotal role in alleviating the pervasive global water scarcity [11,12,13]. RO relies on the application of high pressure to force saline water (e.g., brackish water/seawater) via a semipermeable membrane, thereby enabling water molecules to permeate while retaining salts. Recent technological advancements in membrane materials have notably enhanced the energy efficiency, minimized environmental repercussions, and decreased costs associated with this process. A major advantage of RO lies in its capability to furnish a dependable source of potable water in areas where conventional water sources are inadequate, thereby making it a vital component for water-scarce regions. The breakthroughs in membrane material evolution have profoundly affected the desalination performance, primarily through the advancements in nanotechnology and the creation of innovative materials. Traditionally, desalination process relied heavily on polymer membranes, particularly polyamide (PA) thin-film composite membranes, which have maintained a stronghold in the RO market due to their judicious balance of permeability and selectivity. Notwithstanding, these membranes frequently encounter issues such as fouling, diminished permeability, and chemical instability, which impede their efficiency and durability in desalination applications [6,14,15].

Recent advancements in water treatment and desalination have led to the emergence of novel membrane technologies, including forward osmosis (FO), membrane distillation (MD), and osmotically assisted RO (OARO) which address diverse environmental and societal needs. FO utilizes osmotic pressure to draw water through a semi-permeable membrane, effectively concentrating solutes while minimizing energy consumption. MD, on the other hand, leverages the temperature difference between two sides of a hydrophobic membrane to facilitate the vaporization and subsequent condensation of water, offering a promising solution for desalination with lower energy requirements. OARO combines the principles of both RO and FO to enhance water recovery and reduce energy usage, making it particularly effective for high-salinity brines [16,17].

This article aims to explore the advancements and challenges in the development and deployment of membrane-based processes in ZLD and MLD systems, and their integration into sustainable water management practices. It evaluates the potential of commercial and innovative membrane technologies, such as RO, FO, and MD, to address critical issues like brine management, energy efficiency, and resource recovery. By examining these technological innovations alongside practical applications, the article seeks to contribute to the optimization of ZLD and MLD strategies, aligning them with global sustainability objectives like the United Nations SDG 6.

## 2. Desalination and Brine Treatment Technologies

**Figure 1:**
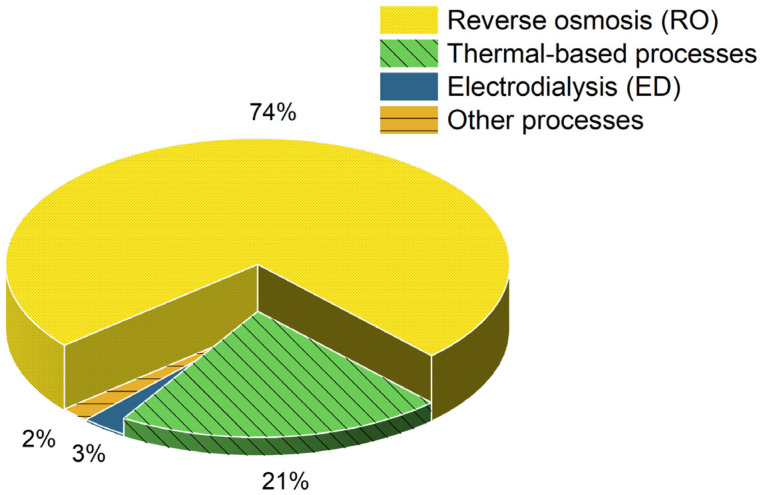
Desalination processes used across the world.

Technologies for desalination can be broadly divided into two groups: membrane-based procedures, which function without phase change, and thermal-based procedures, which entail changes in phase. While membrane-based procedures rely on pressure-driven mechanisms that either facilitate or restrict the transport of particular ions across semi-permeable membranes, thermal-based procedures mimic the natural hydrological cycle of evaporation followed by condensation [18,19,20]. In general, membrane-based techniques rely mostly on electricity, while thermal-based techniques require both heat energy and electrical power. The rates of use of various desalination systems worldwide are shown in Figure 1. According to the data, RO accounts for around 74% of the installed capacity globally, making it the dominant technology in the desalination industry. Thermal-based processes (MSF and MED) account for 21% of the market, while electrodialysis (ED) and other processes, make up the remaining. Because of their greater compactness and energy efficiency, membrane-based desalination techniques are typically chosen over thermal-based ones. However, when it comes to treating high-salinity water, where thermal procedures perform better, membrane-based procedures have limits [11,21].

In recent years, the treatment of brine effluents has become an imperative endeavor, driven by the need to minimize the negative environmental repercussions associated with brine, which often contains concentrated salts and other valuable components. The improper disposal of brine can lead to severe ecological impacts, including harm to marine ecosystems and the degradation of soil and freshwater resources. As a result, innovative approaches for brine management are essential to ensure sustainable desalination practices. The development of multi-disciplinary treatment systems, such as ZLD/MLD, has facilitated the integration of various technologies and processes to optimize brine treatment and enhance resource recovery. These systems not only aim to reduce the volume of brine produced but also to extract valuable materials, such as water, salts, and other nutrients, which can be repurposed for industrial or agricultural applications. Notably, treatment modalities for brine can be categorized into chemical, thermal, membrane-based, and biological processes, which can be effectively hybridized to achieve efficient brine treatment. ZLD systems incorporate an amalgamation of all these treatment technologies, ensuring that nearly all the water is recovered from the brine, leaving behind solid waste. In contrast, MLD systems predominantly employ chemical, membrane-based, and biological processes, focusing on maximizing water recovery while minimizing operational costs [22,23,24,25].

The chemical and biological methods employed in brine treatment can be classified as ion-selective processes and are often used as pre-treatment steps to remove impurities, including organic matter, silica, and heavy metals. Techniques utilized in these processes include chemical precipitation, coagulation, electrocoagulation, ion-exchange, and adsorption. Each of these methods has its advantages and limitations, making it essential to tailor the approach based on the specific characteristics of the brine and the desired recovery outcomes. For instance, chemical precipitation can effectively remove dissolved salts but generate additional waste that requires further treatment. Ion-exchange processes are highly selective and efficient but can be costly due to the need for regenerating the resin. Similarly, adsorption techniques can be employed to capture specific contaminants, yet they may involve considerable operational expenditures [12,26,27,28].

## 3. Membrane-Based Technologies

Membrane processes have gained significant prominence in recent years as a potent means of separating and purifying various substances. These processes rely on the semi-permeable nature of membranes to regulate the passage of molecules and ions across the boundary. In essence, membranes function as highly selective barriers, allowing certain substances to migrate through while rejecting others. Notably, membrane processes have been applied successfully in the treatment of wastewater, desalination, and chemical processing, among other areas. The advantages of membrane processes include reduced energy requirements, improved efficiency, and enhanced product purity compared to traditional methods. As research continues to evolve, the potential applications of membrane processes are anticipated to expand even further [29,30,31,32].

Membrane separation technologies are built upon the foundation of permselective membranes, which exhibit preferential transport of certain components over others. This approach offers several advantages over conventional separation and purification methods, including a cost-effectiveness, operational flexibility, modularity, and reduced energy requirements. In pressure-driven membrane procedures, the transport of mass is facilitated by the pressure gradient generated across the membrane’s feed and permeate compartments. RO is widely employed across the globe to generate potable water. The benefits of utilizing RO membranes are multifaceted, including exceptional water permeability, high-salt rejection, energy recovery capabilities, and conformity to stringent public health/environmental standards [19,27]. Consequently, RO has emerged as a dependable technique capable of effectively eliminating a range of contaminants. Moreover, the desalination sector has achieved significant energy efficiency through RO technology, reportedly requiring approximately 1.8 kWh of energy to produce one cubic meter of water, a notable reduction compared to other desalination methods [33,34].

### 3.1. Reverse Osmosis (RO)

RO desalination process is comprised of four primary subsystems, namely, the pre-treatment unit, high-pressure (HP) pump, membranes/modules, and post-treatment system, each playing a vital role in the overall operation. The RO mechanism relies on a semipermeable membrane-driven process, wherein feed water is subjected to pressure while traversing the membrane, thereby facilitating the separation of the water into two distinct streams. In this process, a high-pressure pump is instrumental in propelling the pre-treated water across the membrane’s surface, thereby optimizing the pressure required for the separation process [3,14,35,36,37,38,39,40].

### 3.2. Forward Osmosis (FO)

The process of FO is a phenomenon characterized by the transfer of molecules from a more concentrated solution to a more dilute solution through a semi-permeable membrane, resulting in the concentration of solutes in the original solution. This process is often utilized in applications where the removal of solutes, such as pollutants or minerals, is desired. The fundamental principle underlying FO is the gradient-driven diffusion of molecules across the membrane, driven by the osmotic pressure difference between the two solutions. In this process, the semipermeable membrane allows the passage of water molecules, while restricting the flow of solutes, thereby creating a concentrative phenomenon. The effectiveness of FO depends on various factors, including the type of membrane utilized, the initial concentration of the solutions, and the operating conditions. As a result, its applications are diverse, spanning from water purification to biotechnology and pharmaceutical industries [10,16,41,42].

### 3.3. Membrane Distillation (MD)

MD is a novel desalination technique that has gained significant attention in recent years. This process leverages the natural ability of hydrophobic membranes to selectively pass through water vapor, while rejecting non-volatile solutes, such as dissolved salts and other impurities. The MD process begins with the pretreatment of a feedwater stream, which is then pumped through a porous hydrophobic membrane. As the hot feedwater stream comes into contact with the membrane, the water molecules undergo rapid diffusion across the membrane, driven by the pressure gradient and the temperature difference. The resulting distillate stream is essentially free from contaminants, making it suitable for various applications, including potable water supply [10,16,43,44,45]. The MD process is energized by an artificially generated temperature disparity between the hot feed water and the cold permeate. In this context, saline water is first heated to a temperature range of 30 °C to 80 °C prior to being fed into the MD module, whereas the permeate is cooled by using the chilly intake saline water, which is maintained at a temperature of less than 20 °C. A higher operating temperature encourages the formation of scaling on the membrane surface, thereby necessitating the addition of an antiscalant to the stream prior to heating. Furthermore, pre-treatment techniques that go beyond the addition of an antiscalant are currently being explored, despite estimates suggesting that MD would demand less chemically- and energy-intensive methods compared to RO [3,46,47,48,49].

### 3.4. Electrodialysis (ED) and Electrodialysis Reversal (EDR)

ED/EDR represent membrane-based technologies that are driven by electrical voltage and have achieved commercial viability in the desalination of brackish water. The fundamental principle of ED involves the selective movement of ions within solutions, utilizing an applied electrical voltage gradient to propel cations and anions in opposing directions through semipermeable membranes. A standard ED configuration consists of a series of alternating cation exchange membranes and anion exchange membranes situated between a cathode and an anode. In this setup, cations migrate towards the negatively charged cathode, whereas anions move towards the positively charged anode, effectively separating freshwater from concentrated brine solutions. In contrast, EDR operates on the same electrochemical principles as ED, with the key distinction being the periodic reversal of direct current voltage, typically occurring three to four times per hour. This voltage reversal facilitates the inversion of ion transport, thereby reducing the occurrence of scaling and fouling [16,50,51,52].

### 3.5. Osmotically Assisted Reverse Osmosis (OARO)

OARO is a novel desalination method that integrates the concepts of FO and RO to enhance efficiency and reduce energy usage. Unlike conventional RO, which requires considerable mechanical pressure to overcome the osmotic pressure of saline water, OARO employs a concentrated draw solution to generate an osmotic gradient that assists in water transport via a semipermeable membrane. This process begins by introducing feed water into a system containing a semipermeable membrane. A concentrated draw solution, with significantly higher osmotic pressure than the feed water, creates a natural gradient that facilitates water transport across the membrane. Unlike conventional systems, where high mechanical pressure is essential, OARO balances osmotic forces and mechanical energy, reducing power consumption while achieving water recovery. Following this step, water extracted into the draw solution undergoes secondary treatment or separation to isolate the fresh product water from the concentrated solution, completing the desalination cycle.

By employing this osmotic pressure to partially counterbalance the required mechanical energy, OARO considerably reduces the energy requirement for desalination, especially when treating very saline water, such as brine streams or saltwater. This innovative method promotes OARO as a possible alternative for energy-efficient desalination and brine concentration. One of the primary advantages of OARO is its capacity to produce significant water recovery rates while running at lower mechanical pressures compared to typical RO systems. This makes it particularly appealing for applications such as ZLD, where minimizing brine waste is crucial.

Additionally, the lower working pressures limit the chance of fouling and scaling on membrane surfaces, which are major issues in conventional RO systems. This not only improves the lifespan of membranes but also minimizes maintenance expenses and operating downtime, thus boosting the economic viability of OARO in industrial and municipal desalination applications [3,53,54,55]. Despite its merits, OARO faces various problems that must be overcome for wider implementation. The maintenance and regeneration of the osmotic draw solution increase complexity to the system and may require additional energy input, offsetting some of the energy savings realized during desalination. Furthermore, OARO relies on sophisticated membrane designs that can endure the combined impacts of hydraulic and osmotic pressures, demanding continual research and development to optimize membrane materials. Another key consideration is the scalability of OARO systems in industrial settings, as ensuring consistent draw solution composition and minimizing water quality fluctuations during operation are essential challenges for its widespread application. Nonetheless, with its ability to minimize energy usage, boost recovery rates, and handle brine management concerns, OARO marks a significant step forward in the evolution of desalination technology [16,56].

### 3.6. Membrane Technologies Evaluation

RO stands out as a reliable and well-established technology. Despite its dominance, several emerging alternatives, such as FO, OARO, and MD), are gaining attention. Each of these technologies offers unique characteristics that require careful evaluation. RO is a membrane-based technology capable of handling feedwaters with a maximum salinity of 70 g/L and achieving a freshwater recovery rate of up to 50%. Its primary advantages include energy efficiency and versatility across a variety of feedwaters. RO has a strong commercial presence and is widely used in full-scale applications. However, it is not without its drawbacks; challenges such as fouling and scaling can impede performance and necessitate frequent maintenance. FO operates on a hydraulic pressure-driven principle, allowing it to process feedwaters with higher levels of suspended solids. It boasts a maximum feed salinity of 200 g/L and an impressive freshwater recovery rate of 98%. The technology is energy-efficient, does not require feed pressure, and has lower fouling and scaling issues compared to RO. Nevertheless, FO faces limitations, including a lack of a universal solution and the necessity for effective draw solution recovery. Currently, it is in the emerging stage, primarily at lab and pilot scales.

OARO is another membrane-based technology that can handle a maximum feed salinity of 140 g/L and achieve up to 72% freshwater recovery. Like FO, it operates without the need for feed pressure and exhibits low fouling and scaling issues. However, like FO, it lacks a universal solution and requires multiple stages for optimal performance. OARO is also in the emerging stage, primarily at lab and pilot scales. MD is distinguished by its ability to handle feedwaters with a maximum salinity of 350 g/L and achieve a freshwater recovery rate of 90%. The reported TDS value of 350 g/L for MD corresponds to experimental studies testing operation near saturation under controlled conditions. However, such conditions may pose significant challenges in practical applications, including severe risks of fouling and scaling due to precipitation [3,57]. Therefore, lower concentrations are typically preferred for sustained operation. MD operates without feed pressure requirements and utilizes low-grade heat or waste heat, which enhances its energy efficiency. However, MD faces challenges such as membrane wetting, low membrane flux, and poor thermal efficiency, which can affect its overall effectiveness. This technology is currently in the emerging stage as well. ED/EDR can manage feedwaters with salinity levels up to 200 g/L, achieving a maximum freshwater recovery of 86%. This technology is notable for its low fouling and scaling issues and its ability to treat brine containing silica. However, it is susceptible to organic fouling, which can hinder its performance. ED/EDR is established as a commercial solution in full-scale applications. For ED/EDR, the previously reported maximum TDS value was based on extrapolated limits under idealized laboratory conditions/multi-stage operation and may not fully represent practical operational capabilities [3,58,59]. A summary of the membrane technologies evaluation is presented in Table 1.

## 4. Membrane Issues

### 4.1. Fouling

Membrane blockage, a collective phenomenon known as ‘fouling’, occurs when materials accumulate on the membrane’s surface or within its pores, thereby compromising membrane flow and necessitating the replacement of membranes and increasing energy expenditures. This process is widely employed for removing substantial amounts of organic compounds, dissolved solids, which is why membrane-based processes are often preferred for these types of applications. Nonetheless, the prevalence of membrane fouling poses significant challenges in water desalination and treatment operations, particularly in cases where water contains high concentrations of naturally occurring organic/inorganic matter [60,61]. The performance of a membrane system is influenced by various solution characteristics, including temperature, pH, membrane flux, foulant content, which in turn determines the optimal configuration and film-forming rate [3,60,62,63]. Membrane fouling can manifest through diverse mechanisms, including the precipitation of low-solubility salts owing to chemical effects, surface clogging by particulate matter, and biological growth, in addition to anaerobic contamination in aerobic systems and physical contact with the membrane surface. Furthermore, the rate of fouling can vary from a frequency of less than once a week to once a year, depending on variables such as pretreatment, system recovery, and feed water characteristics, as well as operating conditions like feed flow velocity and intermittent cycling [22,64,65,66,67,68].

### 4.2. Scaling

In various aquatic systems, the prevailing ions in ordinary water are characterized by considerable sizes, comprising sulfate, calcium, and bicarbonate. Conversely, the solubility limitations of calcite and gypsum are often superseded by elevated levels of saturation in water desalination processes involving membranes undergoing increased rates of recovery. Notably, the most widespread salts responsible for scale formation on the surface of membranes are CaCO_3_, calcium sulphate (CaSO_4_), and silica, leading to the development of hardness-inducing scales. Consequently, to circumvent scaling, it is essential to modify the chemical composition of water to facilitate precipitation prevention, which necessitates operating RO systems at recoveries below a critical threshold to avoid the onset of scaling. In membrane processes, the formation of mineral deposits, which is also referred to as membrane fouling or precipitation, occurs when the ionic product of a sparingly soluble salt exceeds its equilibrium solubility in condensed water. This phenomenon, known as ‘membrane scaling’, can lead to the precipitation of hard scales, which tend to accumulate near and atop the surfaces of the membrane. The gradual accumulation of these mineral deposits can significantly impede membrane function, resulting in reduced flow rates and a substantial shortening of membrane lifespan. Among the most common salt deposits associated with membrane scale formation in surface and groundwater desalination are CaCO_3_ and calcium sulfate dihydrate (gypsum) [10,16,60,69,70,71].

### 4.3. Pore Wetting

Pore wetting is a critical issue that can significantly affect the performance of membrane-based systems. This phenomenon occurs when liquid infiltrates and fills the pores of a membrane, which is particularly problematic in hydrophobic membranes designed to repel water. When the pores become wetted, the permeability of the membrane is substantially reduced, and the resistance to flow increases. This not only diminishes the efficiency of the system but also escalates operational costs due to the need for higher pressure to maintain desired flux levels. Pore wetting can be especially detrimental in applications such as MD or gas separation, where the integrity of the hydrophobic barrier is essential for effective separation processes [16,70]. To address the challenges posed by pore wetting, significant research efforts have been directed toward the development of advanced membrane materials. One promising approach involves the creation of hybrid membranes that combine hydrophilic and hydrophobic properties. These membranes are designed to resist pore wetting while maintaining high flux rates, thereby improving overall performance and system reliability. By carefully tuning the surface chemistry and structural characteristics of these hybrid membranes, researchers aim to achieve a delicate balance between wetting resistance and permeability. Additionally, surface modification techniques, such as grafting hydrophilic polymers or applying nanoscale coatings, have shown potential in enhancing the wetting resistance of existing membrane materials without compromising their functionality [3,70,72].

### 4.4. Concentration Polarization

Concentration polarization is a prevalent issue in membrane-based systems that arises when solutes accumulate near the membrane surface during separation processes. This accumulation creates a concentration gradient, leading to reduced efficiency and increased osmotic pressure, particularly in applications involving high-salinity feeds, such as desalination or brine treatment. As solutes build up near the membrane, the driving force for separation diminishes, and the system’s performance is compromised. Over time, this phenomenon can exacerbate fouling and scaling issues, further hindering operation. In extreme cases, concentration polarization may lead to irreversible damage to the membrane, reducing its lifespan and increasing maintenance costs. To mitigate the effects of concentration polarization, researchers and engineers have developed various strategies to enhance mixing and promote uniform solute distribution near the membrane surface. One effective approach involves optimizing flow patterns within membrane modules, which can disrupt the boundary layer where solute accumulation occurs. For instance, crossflow configurations are commonly employed to improve mixing and reduce the concentration gradient. Additionally, advanced membrane module designs, such as spiral-wound or hollow fiber configurations, have been tailored to minimize concentration polarization by maximizing turbulence and flow distribution across the membrane surface. These design innovations play a crucial role in maintaining system efficiency and preventing performance degradation over time [10,44,56].

To overcome these fouling and scaling, development of anti-fouling membranes using sophisticated materials and surface coatings can assist lower fouling rates. Additionally, establishing pre-treatment methods to eliminate potential foulants before water enters the membrane can be useful [10]. Another key difficulty is pore wetting. Research into hybrid membranes that mix hydrophilic and hydrophobic qualities may provide a solution, allowing for increased resistance to wetting while maintaining high flux rates [70,73].

Concentration polarization is yet additional problem, occurring when solutes collect near the membrane surface, resulting to lower efficiency and higher osmotic pressure. This effect is particularly apparent in high-salinity feeds. To minimize concentration polarization, utilizing flow patterns that increase mixing can be useful. Additionally, optimizing membrane module design, such as employing spiral wound or hollow fiber designs, can assist lessen this effect [10,16,26,74,75,76]. The challenges and potential solutions in membrane technology are summarized in Table 2.

## 5. Zero/Minimal Liquid Discharge (ZLD/MLD) Strategies

### 5.1. Concept

ZLD/MLD approaches have gained increased recognition in recent years, as they offer a vital contribution to the mitigation of hydrological imbalances and the conservation of valuable water resources. Notably, the incorporation of these strategies into industrial processes has been instrumental in reducing the environmental footprint of various sectors, such as the manufacturing and energy production industries. By implementing ZLD technologies, companies can significantly minimize the quantity of wastewater generated and, consequently, alleviate the pressure on municipal water treatment facilities. Recent developments in ZLD/MLD strategies have centered on mitigating the environmental impact of saline wastewater disposal while concurrently valorizing valuable products. To extract multiple valuable resources, including water, salts/minerals, energy, and metals, a range of technologies can be employed either in isolation or as components of integrated ZLD/MLD systems. Notably, hybrid approaches have gained prominence due to their capacity to facilitate the simultaneous recovery of multiple resources while reducing overall energy consumption, subsequently decreasing costs and carbon emissions. Furthermore, the symbiosis of salinity gradient power processes with desalination operations enables interconnected energy recovery, potentially leading to reduced desalination costs. Various desalination technologies are employed in ZLD systems, serving a dual purpose of potable water generation and integrated wastewater treatment [6,10,35,77,78].

### 5.2. Background-History

Water with exceptionally high purity can be derived from ZLD systems, rendering it suitable for various applications, encompassing both residential and industrial sectors. In addition to water, the condensed solid salt byproduct can be marketed, recycled for industrial utilization, or handled in an environmentally sustainable manner. When these technologies are carefully selected, it is feasible to produce multiple high-purity solids rather than a mixed solid. ZLD systems first emerged in the U.S. during the 1970s, specifically in power generation facilities situated along the Colorado River in response to the increasing salinity levels in the waterway. Currently, a considerable proportion of ZLD systems are operational within the U.S., with the majority of these installations being located within the country’s borders. In contrast to the ZLD strategy, which may be a viable option for certain industries, it is essential to identify cost-effective alternatives for industries situated in low-income countries. The MLD emerges as a highly promising and cost-efficient solution for manufacturing industries, contingent upon the fulfillment of legal and environmental prerequisites. Notably, the MLD strategy is akin to the ZLD approach in its reliance on similar technologies, however, the technologies involved typically comprise membrane-based configurations, which have been hybridized to reclaim as much as 95% of freshwater. A notable exemplar of the MLD strategy is the General Motors (G.M.) vehicle production facility in San Luis Potosi, Mexico, which commenced operations in 2008. This facility, which churns out 160,000 vehicles annually, is situated in an arid region approximately 250 miles northwest of Mexico City. By integrating various membrane-based technologies, the plant is capable of converting up to 90% of its waste into water, thereby enabling the release of less than 10% of wastewater into adjacent evaporation ponds [5,10,16,79,80].

### 5.3. ZLD/MLD Configuration

**Figure 2:**
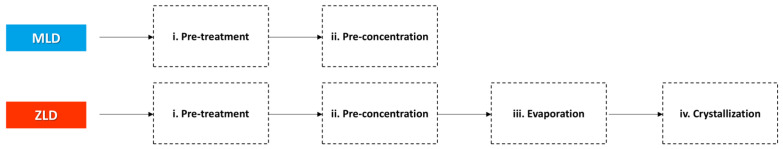
The ZLD and MLD configurations.

When it comes to setting up ZLD/MLD systems, a typical ZLD setup has four main steps: (i) pre-treatment, (ii) pre-concentration, (iii) evaporation, and (iv) crystallization. The first step, pre-treatment, uses various approaches like membrane-based processes and biological-chemical procedures. Its goals are twofold: to get rid of impurities that could hinder the next steps-especially in cleaning the brine—and to extract valuable metals, like precious ions. Generally, if the brine is purer, there is less need for extensive pretreatment. In the second step, membrane-based processes help recover water and cut down on wastewater. This part is significant for cost savings, as it lightens the burden on the following, more expensive steps. The last two steps aim to maximize water recovery, aiming for as much as 100%, minimize the brine left over, completely eliminate saline wastewater, and create one or more solid salt products, mainly using heat-based processes. On the other hand, MLD systems only have two steps: pre-treatment and pre-concentration, with a goal to recover about 95% of the water. Figure 2 illustrates the ZLD/MLD configurations [10,81,82].

### 5.4. Factors Influencing the Adoption of the ZLD/MLD Systems

A complex array of drivers underpinning the widespread implementation of ZLD/MLD systems, each aligned with industrial, economic, and environmental priorities. These catalysts can be categorized as follows:

- Regulatory compliance requirements: Increasingly strict regulatory frameworks governing wastewater discharge, enforced by national and regional authorities, compel industries to adopt advanced treatment methodologies. ZLD/MLD systems offer a pathway to regulatory adherence by ensuring complete elimination of effluent discharge and enabling thorough wastewater purification prior to reuse or safe disposal.
- Escalating water resource constraints: Intensifying global water scarcity, coupled with heightened competition for freshwater reserves, has amplified the urgency for efficient water stewardship. Industries operating in arid regions or those reliant on limited freshwater supplies increasingly adopt ZLD/MLD technologies to optimize water recovery, reduce dependency on external sources, and maintain operational sustainability.
- Commitment to ecological sustainability: The global industrial sector is transitioning toward environmentally responsible practices. ZLD/MLD systems epitomize this shift by significantly reducing water consumption, mitigating aquatic ecosystem contamination, and protecting natural water resources. Their adoption not only reflects environmental accountability but also elevates corporate reputation by enhancing public perception as environmentally responsible entities.
- Material recovery opportunities: ZLD/MLD infrastructure facilitates the extraction and reuse of valuable substances, including metals/minerals, from industrial effluents. This conversion of wastewater into reusable resources minimizes waste generation while generating economic returns, thereby supporting circular economy principles through waste valorization.
- Long-term economic viability: Although ZLD/MLD implementation requires substantial upfront investments, its operational benefits, including reduced freshwater procurement costs, energy efficiency, and revenue from recovered resources, yield significant financial advantages over time. These systems thus represent a strategic balance between initial expenditure and enduring economic gains.
- Risk reduction and legal safeguards: Improper wastewater disposal poses substantial risks to public health, ecological stability, and regulatory compliance. ZLD/MLD systems mitigate these concerns by eliminating discharge through comprehensive contaminant removal, thereby reducing potential legal liabilities and reputational damage associated with environmental violations.
- Innovations in treatment technologies: Progress in engineering solutions, such as enhanced membrane filtration, advanced evaporators, and crystallizer systems, has improved the efficiency and cost-effectiveness of ZLD/MLD processes. These technological advancements have expanded the feasibility of implementation across diverse industrial sectors, enabling scalable and reliable wastewater management solutions.

### 5.5. ZLD/MLD Integrated Systems

Brine effluents have a mix of ions, and getting pure resources from them can be very valuable. To do this, it is necessary to combine different processes in MLD/ZLD systems, as shown in Table 3. The integration of various membrane technologies for ZLD and MLD systems leads to varying cost demands and recovery rates, highlighting their different potential for cost-effective operation in desalination processes.

For instance, the combination of RO and MD offers a 65% recovery rate of water and rubidium, but explicit cost data are unavailable. This integration, while not generally cost-effective for wide-scale desalination, is particularly beneficial in recovering valuable resources, which could be advantageous in specialized applications involving high-value elements like rubidium [83]. When considering the integration of multiple technologies such as RO, high-pressure RO, FO, MD, and OARO, average cost demands range between USD 0.8 and 1.4 per m^3^, with water recovery rates ranging from 78% to 89%. This combination proves to be efficient in both water recovery and energy consumption, making it suitable for large-scale desalination plants while maintaining manageable operational costs [33].

Another promising integration involves adsorption and MD, which achieves a water recovery of 85%, alongside a recovery of 60–98% of rubidium. While no direct cost values are available for this combination, its ability to recover valuable materials like rubidium could help offset the operational costs, enhancing its appeal for resource-focused desalination and brine treatment operations [84]. RO and ED integration shows a slightly lower recovery rate of 77% but provides a balanced energy requirement and operational cost structure, making it economically viable for general desalination applications focused primarily on water recovery [85]. Meanwhile, the integration of ED with ion-exchange processes offers 76.5% recovery of LiCl, with the potential for economically significant applications related to Li recovery, especially considering the growing demand for Li in energy storage technologies [86].

Another combination, RO, NF, and MD, offers a relatively low average cost of USD 0.9 per m^3^, with a water recovery rate of 76.2%. This integration is well-suited for conventional desalination needs while offering competitive recovery and low operational cost, making it a promising solution for both municipal and industrial applications [87]. The integration of RO, MD, and membrane crystallization provides added value by recovering various salts such as NaCl, KCl, and CaCO_3_, in addition to water. The cost for this system averages USD 1.1 per m^3^. The higher operational cost is mitigated by the valuable salt recovery, making it an attractive option for managing brine waste and resource recovery in systems with high salinity or where the recovery of these salts is crucial [88].

MED combined with TVC can achieve over 90% recovery, making it one of the most efficient systems for brine minimization and water production. However, the variability in the operational costs of this system requires further optimization to enhance its accessibility for large-scale desalination applications [89]. Lastly, the integration of MD and NF holds potential for recovering Li, which can be vital for industrial applications, but cost data remain unspecified. This technology’s ability to recover valuable resources while producing water positions it as a potential candidate for specialized resource recovery and desalination processes [90].

These technologies, when analyzed for both cost-effectiveness and recovery potential, reveal a broad spectrum of solutions that cater to varying desalination needs. While higher recovery rates of precious resources generally come with increased complexity and higher costs, simpler water recovery systems tend to offer a more cost-effective solution for general desalination. Additionally, incorporating renewable energy into these systems may significantly reduce the carbon footprint and long-term operating costs, enhancing their economic feasibility and potential for commercialization.

Nonetheless, it is worth mentioning that there is no one-size-fits-all solution, since the makeup of brine effluents can differ a lot. Typically, these effluents have high levels of sodium, potassium, chloride, and calcium ions. Because of this, it is best to focus on removing divalent ions like calcium, sulphate, and magnesium along with any chemicals and bioactive compounds. This is usually done with membrane-based methods like microfiltration (MF), ultrafiltration (UF), nanofiltration (NF), and chemical precipitation. The goals here are twofold: (i) to stop scale build-up on the surfaces of other membrane technologies (like FO and MD), and (ii) to produce a stream that can be used for further valuable applications. Next, separating ions like chloride and sodium as well as important metals such as rubidium, lithium, can be done using various processes. Once the streams are purified, they can be concentrated further through membrane-based processes (i.e., MD, FO) or evaporation processes (i.e., MSF, MED). In ZLD systems, crystallization technologies can be used to form solids. Additionally, it is important to dispose of any leftover concentrated brine in a safe and environmentally friendly manner, especially in MLD systems. One good option is to mix the residual effluent into building materials.

### 5.6. Advantages of Implementing ZLD/MLD Systems

The advantages of adopting ZLD/MLD systems include:

- Environmental sustainability and climate resilience: ZLD/MLD systems contribute to ecological preservation by promoting onsite wastewater reuse, thereby reducing effluent volumes and lowering associated waste management expenditures. This approach supports climate resilience by diminishing water extraction demands and curbing energy-intensive treatment processes.
- Operational cost reduction: By minimizing freshwater procurement costs and reducing reliance on external water sources, ZLD/MLD technologies enhance economic efficiency. Additionally, decreased dependency on supplementary wastewater treatment infrastructure reduces operational expenditures and mitigates public health risks linked to inadequate effluent management.
- Lowered ecological footprint: The adoption of ZLD/MLD systems reduces greenhouse gas emissions by limiting transportation needs for offsite wastewater disposal. Concurrently, it decreases road traffic hazards in local communities and enhances overall environmental performance, thereby reducing exposure to regulatory penalties and future compliance uncertainties.
- Material recovery and valorization: ZLD/MLD processes enable the extraction of reusable materials, such as (NH_4_)_2_SO_4_ for agricultural fertilizers and NaCl for de-icing applications. This resource valorization aligns circular economy principles, transforming waste into economically viable products while minimizing environmental degradation.
- Comprehensive effluent elimination: ZLD/MLD systems achieve near-complete elimination of liquid discharge by redirecting wastewater to offsite treatment facilities, deep-well injection, or thermal destruction methods. While this approach prevents contamination of aquatic ecosystems, it may introduce elevated operational costs in certain scenarios.
- Pollution control and water integrity: Through stringent onsite wastewater reduction strategies and source decontamination practices, ZLD/MLD systems minimize pollutant generation. This ensures the purity of primary water streams, safeguarding water quality for subsequent industrial or municipal applications.

## 6. Challenges, Unfilled Knowledge Flaws, and Future Potentialities in the ZLD/MLD Systems

For desalination systems to be both environmentally and economically feasible, reducing energy consumption and carbon footprints is crucial. Optimizing energy use is necessary to address both the ecological and cost implications of desalination. Membrane technologies exhibit varying energy demands, which influence their associated CO_2_ emissions. Utilizing the energy demands delineated in Table 1, the CO_2_ emissions for all technologies are computed based on power derived from conventional energy sources. RO, with energy requirements of 2–6 kWh/m^3^, typically leads to emissions of 0.9–2.7 kg CO_2_/m^3^. Similarly, FO, with energy usage of 0.8–13 kWh/m^3^, generates emissions ranging from 0.36 to 5.85 kg CO_2_/m^3^, while OARO requires 6–19 kWh/m^3^, leading to emissions between 2.7 and 8.6 kg CO_2_/m^3^. MD, with the highest energy demand of 39–67 kWh/m^3^, results in emissions of 17.6 to 30.15 kg CO_2_/m^3^. Finally, ED/EDR technologies consume 7–15 kWh/m^3^, contributing emissions in the range of 3.15 to 6.75 kg CO_2_/m^3^. However, the type of energy used—high-grade versus low-grade—affects feasibility and efficiency. Developing hybrid systems that can utilize both high-grade and low-grade energy successfully can boost overall energy efficiency. For example, integrating thermal desalination with photovoltaic solar energy can maximize resource use [91,92,93].

As previously discussed, advancements in membrane technology promise to significantly enhance the efficiency and reliability of ZLD/MLD systems. To address membrane-related challenges, innovative membranes have been improved by incorporating materials such as graphene oxide and chitosan onto their surfaces [94,95]. These materials enhance membrane permeability, fouling resistance, and overall durability. Additionally, advanced membrane types with superior properties, including omniphobic and superhydrophobic characteristics, have been developed [96,97,98]. These membranes repel both water and oil-based contaminants, reducing the potential for fouling and scaling, which are common issues in desalination and wastewater treatment processes. Such innovations contribute to enhanced operational efficiency, longer membrane lifespans, and lower maintenance requirements, making them particularly well-suited for demanding applications like ZLD and MLD systems.

Moreover, implementing optimized cleaning routines alongside these improvements can effectively minimize fouling and ensure sustained performance. Investing in long-term studies and pilot projects is essential to generate valuable data on membrane durability and performance, guiding future developments and commercial applications. However, a significant gap remains in understanding the long-term performance and degradation of advanced membrane materials under diverse operational conditions.

In addition to improving membranes, geochemical modeling is a helpful tool for studying brine and looking into ZLD/MLD systems. This type of modeling can make good predictions about precipitation processes for brines, helping to find the best treatment technologies for different brine wastes. Still, modeling very salty brines poses a big challenge. Right now, most of the work on ZLD/MLD systems has been done at computer and lab levels. Because of this, we suggest trying out pilot-scale investigations and life-cycle assessments (LCAs) to see if these systems are economically practical. LCA is a useful method for figuring out the environmental effects of a product or process while also aiding in designing processes. It offers a way to compare different water supply options and shows how much energy is used for treating brine. Although there has been plenty of analysis of seawater desalination systems through LCAs, there is still a lack of studies focused on treating brine and new desalination technologies.

Achieving actual ZLD using membrane technologies requires overcoming significant challenges, particularly regarding water recovery limitations of standalone processes such as RO. To address this, MD has emerged as a promising technology due to its ability to handle highly concentrated brines that exceed the salinity thresholds of RO. MD, when integrated with RO, forms a complementary system where RO is employed for initial water recovery, and MD further concentrates the brine to near saturation levels. This hybrid approach significantly enhances recovery rates while maintaining operational efficiency. The integration also leverages the strengths of both processes: RO’s high throughput in treating feedwater and MD’s capability to manage extreme salinity levels and utilize low-grade or waste heat sources [83]. In addition to RO and MD integration, advanced configurations involving ED or FO have shown potential in addressing specific ZLD challenges, such as selective ion separation or pre-concentration of feed streams. These configurations create pathways for coupling with crystallizers or evaporation systems to complete the ZLD process effectively. A hybrid approach combining multiple membrane technologies offers a pathway to achieving real ZLD while improving energy efficiency and resource recovery. Such combinations can mitigate limitations inherent to individual processes, paving the way for sustainable brine management and enhanced system reliability in ZLD applications.

## 7. Conclusions

The advancement of ZLD and MLD systems represents a pivotal step in sustainable water and wastewater management across desalination and industrial sectors. This article highlights the essential role of membrane technologies, including RO, FO, MD, OARO and ED/EDR in desalination and brine treatment. While commercial membrane-based systems i.e., RO, ED/EDR, dominate due to their energy efficiency and adaptability, emerging solutions like FO, OARO, and MD are poised to tackle limitations in brine treatment and recovery processes. However, substantial barriers remain. Challenges such as concentration polarization, pore wetting, and low water flux continue to hinder performance and scalability, while issues in material durability and economic feasibility necessitate further innovation. The incorporation of nanotechnology, hybrid configurations, and renewable energy integration offers promising avenues to overcome these hurdles. Moreover, ZLD/MLD strategies stand as vital tools to recover water and valuable resources, aligning with global sustainability goals like SDG 6. By fostering collaboration among stakeholders and investing in research and pilot projects, the future of sustainable desalination appears promising, with the potential to drive resource efficiency and minimize environmental impact globally.

## Figures and Tables

**Table 1:** Membrane technologies evaluation [10].

Process	Max. Feed TDS (g/L)	Max. Water Recovery (%)	Pros	Cons	Energy Demands	Status	Cost (USD/m^3^)
RO	70	50	Energy-efficient	- Issues of scaling and fouling - Not efficient as stand-alone	2–6 kWh/m^3^	Commercial	0.75
FO	200	98	- No feed pressure demands- Low issues of scaling and fouling - Energy-efficient	- Lack of universal solution- Demand for draw solution reclamation	0.8–13 kWh/m^3^	Emerging	0.63
OARO	140	72	- No feed pressure demands- Low issues of scaling and fouling - Energy-efficient	- Lack of universal solution- Demand for multi-stage operation - Demand for draw solution reclamation	6–19 kWh/m^3^	Emerging	2.40
MD	350	90	- No feed pressure demands- Low issues of scaling and fouling - Waste heat and/or low-grade heat valorization	- Membrane wetting - Low membrane flux and poor thermal efficiency	39–67 kWh/m^3^	Emerging	1.17
ED/EDR	200	86	- Low issues of scaling and fouling - Available for treating brine with silica content	Issues of organic fouling	7–15 kWh/m^3^	Commercial	0.85

**Table 2:** Challenges and potential solutions in membrane desalination.

Category	Challenges	Potential Solutions
Fouling and Scaling	Membrane fouling from particles, bacteria, and organic matter; scaling from ion precipitation; both lead to reduced efficiency and increased costs, especially in complex feed streams.	Development of anti-fouling membranes with advanced materials; pre-treatment methods to remove foulants before water enters the membrane.
Pore Wetting	In MD, pores filled with liquid significantly lower permeability and increase flow resistance, particularly in hydrophobic membranes.	Research into hybrid membranes combining hydrophilic and hydrophobic properties to enhance wetting resistance while maintaining high flux rates.
Concentration Polarization	Solutes accumulating near the membrane surface lower efficiency and increase osmotic pressure, especially in high-salinity feeds.	Utilizing flow patterns to enhance mixing; optimizing membrane module designs (e.g., spiral wound or hollow fiber) to reduce concentration polarization effects.

**Table 3:** ZLD/MLD integrations.

ZLD/MLD Integration	Average Cost Demands (USD/ m^3^)	Recovery	Resources Reclaimed	Reference
RO, MD	-	65%	water, rubidium	[83]
RO, high-pressure RO, FO, MD, OARO	0.8–1.4	78–89%	water	[33]
Adsorption, MD	-	85% (water) 60–98% (Rb)	water, rubidium	[84]
RO, ED	-	77%	water	[85]
ED, ion-exchange	-	76.5% (LiCl)	water, LiCl	[86]
RO, NF, MD	0.9	76.2% (water)	water	[87]
RO, MD, membrane crystallization	1.1	N/A	water, sodium chloride, potassium chloride, calcium carbonate	[88]
MED, thermal vapor compression	Variable	>90%	water, mixed salt	[89]
MD, NF	-	-	water, lithium	[90]

## References

[1] Mujtaba I.M., Sowgath M.T. (2022). Desalination Technologies: Design and Operation.

[2] Hoek E.M.V., Jassby D., Kaner R.B., Wu J., Wang J., Liu Y., Rao U. (2021). Sustainable Desalination and Water Reuse.

[3] Panagopoulos A., Haralambous K.J., Loizidou M. (2019). Desalination Brine Disposal Methods and Treatment Technologies—A Review. Sci. Total Environ..

[4] Kucera J. (2015). Reverse Osmosis: Industrial Processes and Applications.

[5] Panagopoulos A., Haralambous K.-J. (2020). Minimal Liquid Discharge (MLD) and Zero Liquid Discharge (ZLD) Strategies for Wastewater Management and Resource Recovery—Analysis, Challenges and Prospects. J. Environ. Chem. Eng..

[6] Panagopoulos A. (2021). Water-Energy Nexus: Desalination Technologies and Renewable Energy Sources. Environ. Sci. Pollut. Res..

[7] Arora R., Mutz D., Mohanraj P. (2023). Innovating for The Circular Economy.

[8] Nestorovic M., Radicevic T.D., Belgrade F., Sad N. Transition to Circular Economy. Proceedings of the 41st International Scientific Conference on Economic and Social Development.

[9] Ghangrekar M.M. (2023). Wastewater to Water: Principles, Technologies and Engineering Design.

[10] Panagopoulos A. (2022). Brine Management (Saline Water & Wastewater Effluents): Sustainable Utilization and Resource Recovery Strategy through Minimal and Zero Liquid Discharge (MLD & ZLD) Desalination Systems. Chem. Eng. Process. Process Intensif..

[11] Panagopoulos A., Haralambous K.J. (2020). Environmental Impacts of Desalination and Brine Treatment—Challenges and Mitigation Measures. Mar. Pollut. Bull..

[12] Rosen M., Farsi A. (2022). Sustainable Energy Technologies for Seawater Desalination.

[13] Gude V.G. (2018). Emerging Technologies for Sustainable Desalination Handbook.

[14] Ismail A.F., Khulbe K.C., Matsuura T. (2018). Reverse Osmosis.

[15] Dhakal N. (2017). Controlling Biofouling in Seawater Reverse Osmosis Membrane Systems.

[16] Panagopoulos A., Giannika V. (2022). Decarbonized and Circular Brine Management/Valorization for Water & Valuable Resource Recovery via Minimal/Zero Liquid Discharge (MLD/ZLD) Strategies. J. Environ. Manag..

[17] Valdés H., Saavedra A., Flores M., Vera-Puerto I., Aviña H., Belmonte M. (2021). Reverse Osmosis Concentrate: Physicochemical Characteristics, Environmental Impact, and Technologies. Membranes.

[18] Theodore L., Dupont R.R. (2021). Introduction to Desalination: Principles and Calculations.

[19] Salinas-Rodríguez S.G., Schippers J.C., Amy G.L., Kim I.S., Kennedy M.D. (2021). Seawater Reverse Osmosis Desalination: Assessment and Pre-Treatment of Fouling and Scaling.

[20] Cohen Y. (2021). Advances in Water Desalination Technologies.

[21] Panagopoulos A., Giannika V. (2022). Comparative Techno-Economic and Environmental Analysis of Minimal Liquid Discharge (MLD) and Zero Liquid Discharge (ZLD) Desalination Systems for Seawater Brine Treatment and Valorization. Sustain. Energy Technol. Assess..

[22] Panagopoulos A. (2021). Energetic, Economic and Environmental Assessment of Zero Liquid Discharge (ZLD) Brackish Water and Seawater Desalination Systems. Energy Convers. Manag..

[23] Panagopoulos A. (2022). Study and Evaluation of the Characteristics of Saline Wastewater (Brine) Produced by Desalination and Industrial Plants. Environ. Sci. Pollut. Res..

[24] Salamanca M., Peña M., Hernandez A., Prádanos P., Palacio L. (2023). Forward Osmosis Application for the Removal of Emerging Contaminants from Municipal Wastewater: A Review. Membranes.

[25] Cifuentes-Cabezas M., García-Suarez L., Soler-Cabezas J.L., Cuartas-Uribe B., Álvarez-Blanco S., Mendoza-Roca J.A., Vincent-Vela M.C. (2023). Feasibility of Forward Osmosis to Recover Textile Dyes Using Single Salts and Multicomponent Draw Solutions. Membranes.

[26] Panagopoulos A. (2022). Techno-Economic Assessment of Zero Liquid Discharge (ZLD) Systems for Sustainable Treatment, Minimization and Valorization of Seawater Brine. J. Environ. Manag..

[27] Ayoub G.M., Korban L., Al-Hindi M., Zayyat R. (2019). Brackish Water Desalination: An Effective Pretreatment Process for Reverse Osmosis Systems. Water Air Soil Pollut..

[28] Nascimento T.A., Fdz-Polanco F., Peña M. (2020). Membrane-Based Technologies for the Up-Concentration of Municipal Wastewater: A Review of Pretreatment Intensification. Sep. Purif. Rev..

[29] Panagopoulos A. (2021). Beneficiation of Saline Effluents from Seawater Desalination Plants: Fostering the Zero Liquid Discharge (ZLD) Approach—A Techno-Economic Evaluation. J. Environ. Chem. Eng..

[30] Panagopoulos A., Giannika V. (2024). Techno-Economic Analysis (TEA) of Zero Liquid Discharge (ZLD) Systems for Treatment and Utilization of Brine via Resource Recovery. Chem. Eng. Process. Process Intensif..

[31] Shemer H., Wald S., Semiat R. (2023). Challenges and Solutions for Global Water Scarcity. Membranes.

[32] Zuo J., Chow C.A., Dumée L.F., Prince A.J. (2022). A Zero-Brine Discharge Seawater Desalination Using a Pilot-Scale Membrane Distillation System Integrated with Crystallizer. Membranes.

[33] Panagopoulos A. (2021). Techno-Economic Assessment of Minimal Liquid Discharge (MLD) Treatment Systems for Saline Wastewater (Brine) Management and Treatment. Process Saf. Environ. Prot..

[34] Panagopoulos A. (2020). A Comparative Study on Minimum and Actual Energy Consumption for the Treatment of Desalination Brine. Energy.

[35] Panagopoulos A. (2023). Zero Liquid Discharge and Minimal Liquid Discharge Strategies for Sustainable Saline Wastewater (Brine) Management and Valorization. Resource Recovery in Industrial Waste Waters.

[36] Hong S., Park K., Kim J., Alayande A.B., Kim Y. (2023). Seawater Reverse Osmosis (SWRO) Desalination—Energy Consumption in Plants, Advanced Low-Energy Technologies, and Future Developments for Improving Energy Efficiency.

[37] Mohanadas D., Nordin P.M.I., Rohani R., Dzulkharnien N.S.F., Mohammad A.W., Mohamed Abdul P., Abu Bakar S. (2023). A Comparison between Various Polymeric Membranes for Oily Wastewater Treatment via Membrane Distillation Process. Membranes.

[38] Liang J., Huang H., Zhang H., Wu Y., Zhuang Y. (2023). Preparation of Thin Film Composite (TFC) Membrane with DESPs Interlayer and Its Forward Osmosis (FO) Performance for Organic Solvent Recovery. Membranes.

[39] Tan Y.Z., Alias N.H., Aziz M.H.A., Jaafar J., Othman F.E.C., Chew J.W. (2023). Progress on Improved Fouling Resistance-Nanofibrous Membrane for Membrane Distillation: A Mini-Review. Membranes.

[40] Tomczak W., Gryta M. (2021). Membrane Distillation of Saline Water Contaminated with Oil and Surfactants. Membranes.

[41] Abdullah N., Tajuddin M.H., Yusof N. (2018). 10—Forward Osmosis (FO) for Removal of Heavy Metals.

[42] Ng D.Y.F., Chen Y., Dong Z., Wang R. (2019). Membrane Compaction in Forward Osmosis Process. Desalination.

[43] Panagopoulos A., Giannika V. (2024). A Comprehensive Assessment of the Economic and Technical Viability of a Zero Liquid Discharge (ZLD) Hybrid Desalination System for Water and Salt Recovery. J. Environ. Manag..

[44] Skuse C., Gallego-Schmid A., Azapagic A., Gorgojo P. (2020). Can Emerging Membrane-Based Desalination Technologies Replace Reverse Osmosis?. Desalination.

[45] Im B.-G., Francis L., Santosh R., Kim W.-S., Ghaffour N., Kim Y.-D. (2022). Comprehensive Insights into Performance of Water Gap and Air Gap Membrane Distillation Modules Using Hollow Fiber Membranes. Desalination.

[46] Abdel-Karim A., Leaper S., Skuse C., Zaragoza G., Gryta M., Gorgojo P. (2021). Membrane Cleaning and Pretreatments in Membrane Distillation—A Review. Chem. Eng. J..

[47] Gontarek-Castro E., Castro-Muñoz R., Lieder M. (2022). New Insights of Nanomaterials Usage toward Superhydrophobic Membranes for Water Desalination via Membrane Distillation: A Review. Crit. Rev. Environ. Sci. Technol..

[48] Yadav A., Singh K., Panda A.B., Labhasetwar P.K., Shahi V.K. (2021). Membrane Distillation Crystallization for Simultaneous Recovery of Water and Salt from Tannery Industry Wastewater Using TiO_2_ Modified Poly(Vinylidene Fluoride-Co-Hexafluoropropylene) Nanocomposite Membranes. J. Water Process Eng..

[49] Baroud T.N. (2023). Tuning PVDF Membrane Porosity and Wettability Resistance via Varying Substrate Morphology for the Desalination of Highly Saline Water. Membranes.

[50] Zhao D., Lee L.Y., Ong S.L., Chowdhury P., Siah K.B., Ng H.Y. (2019). Electrodialysis Reversal for Industrial Reverse Osmosis Brine Treatment. Sep. Purif. Technol..

[51] Guo H., Kim Y. (2021). Membrane Scaling in Electrodialysis Fed with High-Strength Wastewater. Environ. Eng. Sci..

[52] Kurihara M. (2021). Current Status and Future Trend of Dominant Commercial Reverse Osmosis Membranes. Membranes.

[53] Askari M., Liang C.Z., Choong L.T., Chung T.S. (2021). Optimization of TFC-PES Hollow Fiber Membranes for Reverse Osmosis (RO) and Osmotically Assisted Reverse Osmosis (OARO) Applications. J. Memb. Sci..

[54] Foo K., Liang Y.Y., Lau W.J., Khan M.M.R., Ahmad A.L. (2023). Performance of Hypersaline Brine Desalination Using Spiral Wound Membrane: A Parametric Study. Membranes.

[55] Cifuentes-Cabezas M., Álvarez-Blanco S., Mendoza-Roca J.A., Vincent-Vela M.C., Gozálvez-Zafrilla J.M. (2023). Theoretical Model for the Prediction of Water Flux during the Concentration of an Olive Mill Wastewater Model Solution by Means of Forward Osmosis. Membranes.

[56] Shah M.P. (2023). Membrane and Membrane-Based Processes for Wastewater Treatment.

[57] Tunc C.M., Groth A.M. (2011). Sustainable Integrated Membrane Contactor Process for Water Reclamation, Sodium Sulfate Salt and Energy Recovery from Industrial Effluent. Desalination.

[58] Jiang C., Wang Y., Zhang Z., Xu T. (2014). Electrodialysis of Concentrated Brine from RO Plant to Produce Coarse Salt and Freshwater. J. Memb. Sci..

[59] Reig M., Casas S., Aladjem C., Valderrama C., Gibert O., Valero F., Centeno C.M., Larrotcha E., Cortina J.L. (2014). Concentration of NaCl from Seawater Reverse Osmosis Brines for the Chlor-Alkali Industry by Electrodialysis. Desalination.

[60] Saji V.S., Meroufel A.A., Sorour A.A. (2020). Corrosion and Fouling Control in Desalination Industry.

[61] Song W., Lee L.Y., Liu E., Shi X., Ong S.L., Ng H.Y. (2020). Spatial Variation of Fouling Behavior in High Recovery Nanofiltration for Industrial Reverse Osmosis Brine Treatment towards Zero Liquid Discharge. J. Memb. Sci..

[62] Panagopoulos A., Giannika V. (2023). Study on the Water Resources and the Opportunities for Sustainable Desalination & Minimal/Zero Liquid Discharge (MLD/ZLD) Practices in Greece (Eastern Mediterranean). Sustain. Water Resour. Manag..

[63] Panagopoulos A. (2020). Techno-Economic Evaluation of a Solar Multi-effect Distillation/Thermal Vapor Compression Hybrid System for Brine Treatment and Salt Recovery. Chem. Eng. Process. Process Intensif..

[64] Panagopoulos A. (2022). Techno-Economic Assessment and Feasibility Study of a Zero Liquid Discharge (ZLD) Desalination Hybrid System in the Eastern Mediterranean. Chem. Eng. Process. Process Intensif..

[65] Pennington K.L., Cech T.V. (2019). Introduction to Water Resources and Environmental Issues.

[66] Sarvestani A.B., Chogani A., Shariat M., Moosavi A., Kariminasab H. (2021). The Effect of Nanopores Geometry on Desalination of Single-Layer Graphene-Based Membranes: A Molecular Dynamics Study. J. Mol. Liq..

[67] Imdad S., Dohare R.K. (2022). A Critical Review on Heavy Metals Removal Using Ionic Liquid Membranes from The Industrial Wastewater. Chem. Eng. Process. Process Intensif..

[68] Lee T.H., Roh J.S., Yoo S.Y., Roh J.M., Choi T.H., Park H.B. (2020). High-Performance Polyamide Thin-Film Nanocomposite Membranes Containing ZIF-8/CNT Hybrid Nanofillers for Reverse Osmosis Desalination. Ind. Eng. Chem. Res..

[69] Santos A.V., Lin A.R.A., Amaral M.C.S., Oliveira S.M.A.C. (2021). Improving Control of Membrane Fouling on Membrane Bioreactors: A Data-Driven Approach. Chem. Eng. J..

[70] Horseman T., Yin Y., Christie K.S.S., Wang Z., Tong T., Lin S. (2021). Wetting, Scaling, and Fouling in Membrane Distillation: State-of-the-Art Insights on Fundamental Mechanisms and Mitigation Strategies. ACS EST Eng..

[71] Carmona B., Abejón R. (2023). Innovative Membrane Technologies for the Treatment of Wastewater Polluted with Heavy Metals: Perspective of the Potential of Electrodialysis, Membrane Distillation, and Forward Osmosis from a Bibliometric Analysis. Membranes.

[72] Sharma A.K., Juelfs A., Colling C., Sharma S., Conover S.P., Puranik A.A., Chau J., Rodrigues L., Sirkar K.K. (2021). Porous Hydrophobic–Hydrophilic Composite Hollow Fiber and Flat Membranes Prepared by Plasma Polymerization for Direct Contact Membrane Distillation. Membranes.

[73] Wang J., Wu W. (2023). Functional Membranes for High Efficiency Molecule and Ion Transport.

[74] Wei H., Zhao S., Zhang X., Wen B., Su Z. (2021). The Future of Freshwater Access: Functional Material-Based Nano-Membranes for Desalination. Mater. Today Energy.

[75] Zhang N., Song X., Jiang H., Tang C.Y. (2021). Advanced Thin-Film Nanocomposite Membranes Embedded with Organic-Based Nanomaterials for Water and Organic Solvent Purification: A Review. Sep. Purif. Technol..

[76] Nakao T., Miura Y., Furuichi K., Yasukawa M. (2021). Cellulose Triacetate (Cta) Hollow-Fiber (Hf) Membranes for Sustainable Seawater Desalination: A Review. Membranes.

[77] Abounahia N., Shahab A.A., Khan M.M., Qiblawey H., Zaidi S.J. (2023). A Comprehensive Review of Performance of Polyacrylonitrile-Based Membranes for Forward Osmosis Water Separation and Purification Process. Membranes.

[78] Vera-Villalobos H., Riquelme C., Silva-Aciares F. (2023). Use of Alteromonas Sp. Ni1-LEM Supernatant as a Cleaning Agent for Reverse-Osmosis Membranes (ROMs) from a Desalination Plant in Northern Chile Affected by Biofouling. Membranes.

[79] Yu J., Jing W., Liu E., Du S., Cai H., Du H., Wang J. (2023). Effect of Polydopamine/Sodium Dodecyl Sulfate Modified Halloysite on the Microstructure and Permeability of a Polyamide Forward Osmosis Membrane. Membranes.

[80] Abu-Zeid M.A.E.R., Bassyouni M., Fouad Y., Monica T., Sandid A.M., Elhenawy Y. (2023). Experimental and Simulation Study of Solar-Powered Air-Gap Membrane Distillation Technology for Water Desalination. Membranes.

[81] Ju J., Lee S., Kim Y., Cho H., Lee S. (2023). Theoretical and Experimental Analysis of Osmotically Assisted Reverse Osmosis for Minimum Liquid Discharge. Membranes.

[82] Jahan N., Tahmid M., Shoronika A.Z., Fariha A., Roy H., Pervez M.N., Cai Y., Naddeo V., Islam M.S. (2022). A Comprehensive Review on the Sustainable Treatment of Textile Wastewater: Zero Liquid Discharge and Resource Recovery Perspectives. Sustainability.

[83] Naidu G., Jeong S., Johir M.A.H., Fane A.G., Kandasamy J., Vigneswaran S. (2017). Rubidium Extraction from Seawater Brine by an Integrated Membrane Distillation-Selective Sorption System. Water Res..

[84] Choi Y., Ryu S., Naidu G., Lee S., Vigneswaran S. (2019). Integrated Submerged Membrane Distillation-Adsorption System for Rubidium Recovery. Sep. Purif. Technol..

[85] McGovern R.K., Zubair S.M., Lienhard V.J.H. (2014). Hybrid Electrodialysis Reverse Osmosis System Design and Its Optimization for Treatment of Highly Saline Brines. IDA J. Desalination Water Reuse.

[86] Guo Z.Y., Ji Z.Y., Chen Q.B., Liu J., Zhao Y.Y., Li F., Liu Z.Y., Yuan J.S. (2018). Prefractionation of LiCl from Concentrated Seawater/Salt Lake Brines by Electrodialysis with Monovalent Selective Ion Exchange Membranes. J. Clean. Prod..

[87] El-Zanati E., El-Khatib K.M. (2007). Integrated Membrane--Based Desalination System. Desalination.

[88] Creusen R., van Medevoort J., Roelands M., van Renesse van Duivenbode A., Hanemaaijer J.H., van Leerdam R. (2013). Integrated Membrane Distillation-Crystallization: Process Design and Cost Estimations for Seawater Treatment and Fluxes of Single Salt Solutions. Desalination.

[89] Panagopoulos A. (2020). Process Simulation and Techno-Economic Assessment of a Zero Liquid Discharge/Multi-Effect Desalination/Thermal Vapor Compression (ZLD/MED/TVC) System. Int. J. Energy Res..

[90] Park S.H., Kim J.H., Moon S.J., Jung J.T., Wang H.H., Ali A., Quist-Jensen C.A., Macedonio F., Drioli E., Lee Y.M. (2020). Lithium Recovery from Artificial Brine Using Energy-Efficient Membrane Distillation and Nanofiltration. J. Memb. Sci..

[91] Gude V.G. (2018). Renewable Energy Powered Desalination Handbook: Application and Thermodynamics.

[92] Azar A.T., Kamal N.A. (2021). Design, Analysis, and Applications of Renewable Energy Systems.

[93] Alawad S.M., Mansour R.B., Al-Sulaiman F.A., Rehman S. (2023). Renewable Energy Systems for Water Desalination Applications: A Comprehensive Review. Energy Convers. Manag..

[94] Silvestro I., Ciarlantini C., Francolini I., Tomai P., Gentili A., Dal Bosco C., Piozzi A. (2021). Chitosan–Graphene Oxide Composite Membranes for Solid-Phase Extraction of Pesticides. Int. J. Mol. Sci..

[95] Saputra A.M.A., Agustina N., Amran A., Zurnansyah Z., Samnur S., Sujiono E.H. (2022). Synthesis and Characterisation of Graphene Oxide/Chitosan Composite Membranes from Natural Waste. J. Phys. Sci..

[96] Ni T., Lin J., Kong L., Zhao S. (2021). Omniphobic Membranes for Distillation: Opportunities and Challenges. Chin. Chem. Lett..

[97] Prasanna N.S., Choudhary N., Singh N., Raghavarao K.S.M.S. (2023). Omniphobic Membranes in Membrane Distillation for Desalination Applications: A Mini-Review. Chem. Eng. J. Adv..

[98] Chen L.H., Huang A., Chen Y.R., Chen C.H., Hsu C.C., Tsai F.Y., Tung K.L. (2018). Omniphobic Membranes for Direct Contact Membrane Distillation: Effective Deposition of Zinc Oxide Nanoparticles. Desalination.

